# A novel *PAX6* mutation in a large Chinese family with aniridia and congenital cataract

**Published:** 2010-06-22

**Authors:** Fucheng Cai, Jianfang Zhu, Wen Chen, Tie Ke, Fang Wang, Xin Tu, Ying Zhang, Runming Jin, Xiaoyan Wu

**Affiliations:** 1 Pediatrics Department of Union Hospital, Huazhong University of Science and Technology, Wuhan, Hubei, China; 2 Central laboratory of Union Hospital, Huazhong University of Science and Technology, Wuhan, Hubei, China; 3 Ophthalmology Department of Union Hospital, Huazhong University of Science and Technology, Wuhan, Hubei, China; 4 Center for Human Genome Research, College of Life Science and Technology, Huazhong University of Science and Technology, Wuhan, Hubei, China

## Abstract

**Purpose:**

To identify the disease-causing gene in a four-generation Chinese family affected with autosomal dominant aniridia and cataract.

**Methods:**

All patients underwent full ophthalmic examination. For mutation analysis, a partial coding region (exons 5–14) of paired box gene 6 (*PAX6*) was sequenced with DNA from the proband. Single-strand conformation polymorphism analysis for exon 5 of *PAX6* was performed to demonstrate co-segregation of the *PAX6* mutation with aniridia in all family members and the absence of the mutation in the normal controls.

**Results:**

The proband and other patients in the family were affected with aniridia accompanied with congenital cataract. A novel heterozygous *PAX6* mutation in exon 5 (c.475_491del17, p.Arg38ProfsX12) was identified, which was predicted to generate a frameshift and create a premature termination codon. This mutation co-segregated with the affected individuals in the family and did not exist in unaffected family members and 100 unrelated normal controls.

**Conclusions:**

A novel deletion mutation in the *PAX6* gene was identified in a Chinese family with aniridia and congenital cataract. Our study expands the mutation spectrum of *PAX6*.

## Introduction

Barratta [1] first described aniridia in 1818 (OMIM 106210). Although called aniridia (Greek for absence of the iris), this disorder is not just an isolated defect in iris development but is a panocular disorder that involves the lens, optic nerve, cornea, anterior chamber, and retina [2]. Aniridia occurs in the general population at a frequency of approximately 1 in 64,000–96,000, and two-thirds of cases are familial with autosomal dominant inheritance with complete penetrance but variable expressivity [3].

Paired box gene 6 (*PAX6*), a member of the paired box gene family, is located on chromosome 11p13. *PAX6* is divided into 14 exons that span over 22 kb in length [4]. The polypeptide product possesses several functional domains: a paired domain and a homeodomain separated by a linker segment and followed by a COOH-terminal region rich in proline, serine, and threonine [5]. The paired domain, which is encoded by exons 5–7 of *PAX6*, comprises two structurally distinct subdomains, the relatively conserved NH_2_ terminal (NTS) and the variable COOH terminal [6,7]. *PAX6* plays a major role in the organization of the developing eye [3], and various heterozygous mutations in *PAX6* have been identified in patients with aniridia. To date over 20 different *PAX6* mutations, which have been reported in the Human *PAX6* Allelic Variant Database [8], are associated with aniridia and congenital cataract.

In this study, we analyzed the coding sequences of *PAX6* in a large Chinese family and identified a novel frameshift mutation that causes aniridia and cataract.

## Methods

### Patients

We investigated a large family that originated from the central region of China. Seven living patients with aniridia and congenital cataract were identified. Informed consent was obtained from the participants in accordance with the study protocols approved by the ethics committee of Union Hospital of Huazhong University of Science and Technology, Wuhan, China. The proband in this family received a complete ophthalmic evaluation, and the other five subjects (ocular data of II-11 could not be obtained) underwent ocular slit-lamp examination at Union Hospital.

### Mutation screening

Venous blood (5 ml) was collected from the participants, and total human genomic DNA was isolated with the DNA Isolation Kit for Mammalian Blood (Roche Diagnostic Company, Indianapolis, IN). Considering that the *PAX6* mutation is a genetic factor common to hereditary aniridia and at least 20 different mutations of *PAX6* are responsible for both aniridia and congenital cataract, we carried out mutation screening in the *PAX6* gene directly without performing linkage analysis. Because the ten coding exons (exon 5–14) have been thought to be the hot spots for *PAX6* mutation [8], eight pairs of primers (Table 1) were used to amplify these regions. Briefly, amplification was performed in the PTC-200 thermal cycler (MJ Research Inc., Waterdown, MA) in a 25-μl reaction mixture containing 1.5 mM MgCl_2_, 0.2 mM of each dNTP (Qiagen, Hilden, Germany), 0.5 μM primers, 1 U of Taq DNA polymerase (Qiagen), and 50 ng of genomic DNA. PCR was performed as follows: an initial denaturation step was carried out for 3 min at 94 °C, nine cycles of 30 s at 94 °C, 30 s at the respective annealing temperature (see Table 1) and 30 s at 72 °C, followed by the same 27 cycles with a separate annealing temperature (Table 1). Direct bidirectional resequencing of all PCR-amplified products was performed with the BigDye Terminator Cycle Sequencing v3.1 kit (Applied Biosystems, Foster City, CA) and electrophoresed on an ABI PRISM 3730 Genetic Analyzer (Applied Biosystems). Sequencing results from the subjects and *PAX6* consensus sequences from the NCBI human genome database (NM_000280.3) were compared by using BLAST analysis. Mutation description followed the nomenclature recommended by the Human Genomic Variation Society.

**Table 1:** Primers used for polymerase chain reaction amplification and sequencing of *PAX6*.

** **	** **	** **	**Annealing temperature °C**		** **
**Exon**	**Sense primer(5’-3’)**	**Antisense primer (5’-3’)**	**I**	**II**	**Product size (bp)**
5	GGCTGGTGGTCCTGTTGTCCTT	CGAGCCCGAAGTCCCAGAAAG	61.5	55	483
6, 7	AAGCAAGGTCAGCACAAAAATAAATT	GGAGGAGGTAAAGAGGAGAGAGCATT	60.5	54	648
8	TAAGGTTGTGGGTGAGCTGAGATG	GGGAGAGTAGGGGACAGGCAAAGG	61.5	55	315
9	TTTGGTGAGGCTGTCGGGATATAAT	TGCCCAGAGAAATAAAAAGACAGAAA	61.5	55	415
10	TTGGTTGGAGGTAATGGGAGTGG	TGGCAGCAGAGCATTTAGCAGAC	61.5	55	334
11, 12	GGGGCTGGGCTCGACGTAG	GCCACCACCAGCCGCACTTA	65	60	438
13	GGGGCTGTGGCTGTGTGATGT	CCCCAGGGACAAGGAAAGCAA	61.5	55	333
14	CCAAACATGCAAACAAACAGAGGA	TTCCAACTGATATCGTGCCTTCTG	61.5	55	570

### Single-strand conformation polymorphism analysis

The novel variation detected in exon 5 of *PAX6* was further evaluated in 28 available family members as well as normal control subjects by using single-strand conformation polymorphism (SSCP) analysis, as described previously [9]. Briefly, 2 μl of undigested PCR products was mixed with 4 μl of the degenerating loading buffer, denatured at 95 °C for 10 min and immediately placed on ice; then loaded on 5% polyacrylamide gels, and the DNA samples were separated by electrophoresis overnight at 150 V. The DNA bands were visualized by silver staining.

### Sequencing of variants

We sequenced all variants detected by SSCP. Following electrophoresis, the DNA bands of interest were excised, taking care to remove as much excess gel as possible. The gel slices containing the PCR products with 100 μl of dH_2_O were crushed and melted at 65 °C for 4–5 h. PCR was performed as described above. PCR products were sequenced using the BigDye Terminator v3.1 kit and the ABI 3730 sequencer.

## Results

### Clinical findings

**Figure 1:**
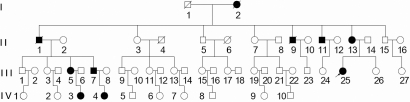
Pedigree of a Chinese family with autosomal-dominant aniridia. Affected males and females are indicated by filled squares and circles, respectively. Normal individuals are shown as empty symbols. Deceased individuals are indicated by slashes (/). The proband is indicated by an arrow.

**Figure 2:**
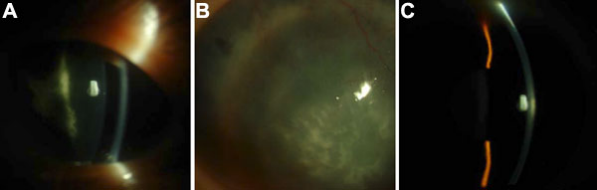
Slit-lamp aspects of the ocular anterior segment of patients. The proband (III-25) exhibits aniridia without iris remnants (**A**); the affected family member (II-13) presents corneal pannus (**B**); the normal subject (III-26) shows a complete iris (**C**).

We identified a large Chinese family with 49 living members in four generations (Figure 1). Bilateral total aniridia, congenital cataract, congenital nystagmus and optic nerve hypoplasia were present in the proband (Figure 2). Ophthalmic manifestations of affected members are listed in Table 2, and all six patients had bilateral total aniridia and congenital posterior subcapsular cataract. Corneal pannus was present in three patients (Figure 2), and congenital nystagmus was present in two patients.

**Table 2:** Ocular phenotypes in six aniridia patients from a Chinese family.

**Patient**	**Age (years)/sex**	**Aniridia**	**Cataracts**	**Corneal pannus**	**Nystagmus**
II-13	54/F	+	+	+	-
III-5	41/F	+	+	+	+
III-7	40/M	+	+*	+	-
III-25**	31/F	+	+	-	+
IV-3	12/F	+	+	-	-
IV-4	12/F	+	+	-	-

The asterisk indicates after left cataract extraction and the double asterisk indicates the proband.

### Mutation analysis

**Figure 3:**
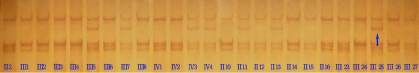
SSCP analysis of *PAX6* mutation in exon 5. Each affected individual has one more band than normal family members. The arrow indicates the extra band of the index patient (III-25).

**Figure 4:**
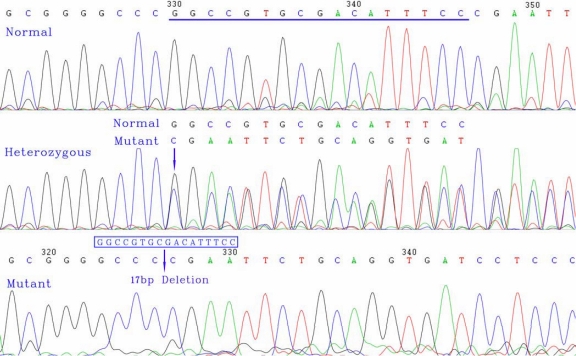
Sequence of the PCR product of exon 5 in *PAX6*. The top chromatogram represents the sequence of a normal family member (normal). The middle chromatogram shows a reading frame shift in the proband (III-25, heterozygous), and the arrow indicates the initiation of the mutation site (beginning of overlapping peaks). The bottom chromatogram exhibits the sequence of the extra band of the SSCP removed from the gel (mutant), and the arrow indicates the location of the mutation.

Direct bidirectional sequencing of *PAX6* in all affected patients revealed a heterozygous 17 bp deletion (c.475_491delGGCCGTGCGACATTTCC) within the paired domain in exon 5. The c.475_491del17 generates a frameshift and a premature termination 12 codons downstream (p.Arg38ProfsX12). SSCP analysis also demonstrated that affected members in the family carried this mutation, but the unaffected members of the family and 100 normal Chinese Han controls did not carry the mutation (Figure 3). In all affected family members, the same heterozygous was confirmed by sequencing the extra bands of PCR-SSCP products (Figure 4). These results suggest that this novel mutation of *PAX6* is not a rare polymorphism but a causative mutation for autosomal-dominant congenital aniridia and congenital cataract in this Chinese family.

## Discussion

In the present study, the identified novel mutation (c.475_491del17) generates a frameshift and a premature termination 12 codons downstream (p.Arg38ProfsX12). The novel mutation in *PAX6* predicted to result in a transcript that is recognized by the nonsense-mediated mRNA decay system [10], leading to a half reduction of the full-length PAX6 protein. This result conforms to genotype–phenotype correlation analysis, suggesting that mutations that introduce a premature termination codon (PTC) into the open reading frame usually result in the aniridia phenotype [11].

The NTS of the paired domain is highly conserved and plays an important role in contacting with the DNA. There is a helix-turn-helix (HTH) unit, containing a β turn and three α helices (helix 1, 2 and 3, residues 23-35, 40-45, and 50-63, respectively) in the NTS; this helix-turn-helix unit makes critical contacts in sugar phosphate backbone, major groove and minor groove. Among those residues, Arg38, Pro39 and Cys40 (Arg38 and Pro39 are in the turn between helices 1 and 2; Cys40 is a part of helix 2), contact with the sugar phosphate backbone of the target DNA [12]. Interestingly, in our patients the deletion mutation (c.475_491del17, p.Arg38ProfsX12) affects residues (Arg38 to Ser43) involving the above-mentioned three amino acids (Arg38, Pro39, and Cys40). Clinical data show that although there are different symptoms in different patients, they have common and severe congenital anomalies in eye development, including the near absence of iris and posterior subcapsular cataract. We evaluated seven unique mutations from the Human *PAX6* Allelic Variant Database [8] that refer to these three amino acids (Table 3) [13-19]. Atchaneeyasakul et al. [16] reported that a novel insertion/deletion mutation (c.474_480del12insGA) detected in a Thai familial aniridia patient affects the three residues completely. The patient had normal best corrected visual acuity but other ocular abnormalities, including partial aniridia, juvenile glaucoma, posterior polar cataracts, corneal pannus, foveal hypoplasia, and ptosis. With the exception of one case [14], it appears that mutations impacting one or two of the three amino acids are associated with an isolated defect in iris development [13,17-19]. In that anomalistic case [14], the patient with complex eye phenotypes was found to have mutations not only in *PAX6* but also in neurofibromin 1 (*NF1*) and orthodenticle homeobox 2 (*OTX2*). In addition, a neighboring mutation (p.41_43delAspIleSer) generates the aniridia phenotype only [20]. We presume the region that the residues (Arg38, Pro39, and Cys40) are in contact with play an important role in the process of optical development, and an anomaly of this region may generate a complex phenotype of ocular organs. Furthermore, mutations that affect residues Asp41-Ser43 (Table 3) [20-24] lead to a consistent aniridia phenotype; two of these mutations result in aniridia accompanied with cataract [23,24]. Although approximate 11% of the mutations in the database do not result in aniridia or cataract, several mutations that refer to the 17 nucleotides deleted in our patients generate both the aniridia and congenital cataract phenotype.

**Table 3:** Mutations impacting on residues (Arg38 to Ser43) cause different phenotypes.

**Genotype**		** **	** **
**cDNA**	**Protein**	**Phenotype**	**Reference**
c.471del9	p.37_39del	Aniridia	[12]
c.474C>T	p.Arg38Trp	Aniridia, microphthalmia, nystagmus, cataract	[13]
c.474delC	p.Arg38GlyfsX16	Aniridia	[14]
c.474_485del12insGA	p.Arg38GlufsX13	Aniridia, glaucoma, cataract, foveal hypoplasia, corneal pannus	[15]
c.476_483del8	p.Pro39HisfsX14	Aniridia	[16]
c.478insCC	p.Cys40ArgfsX15	Iris hypoplasia	[17]
c.482C>A	p.Cys40X	Aniridia	[18]
c.483_491del9	p.41_43delAspIleSer	Aniridia	[19]
c.484A>G	p.Asp41Gly	Iris hypoplasia, nystagmus	[20]
c.486delA	p.Ile42PhefsX12	Aniridia, glaucoma	[21]
c.487T>G	p.Ile42Ser	Aniridia, nystagmus, congenital cataract	[22]
c.489T>C	p.Ser43Pro	Aniridia, cataract, nystagmus	[23]

This study identified a novel deletion mutation of *PAX6* in a Chinese family with aniridia and congenital cataract. This finding expands the mutation spectrum of *PAX6* and is useful and valuable for genetic counseling and prenatal diagnosis in families with aniridia accompanied with congenital cataract.
